# Lipids at the crossroad: Shaping biological membranes heterogeneity defines trafficking pathways

**DOI:** 10.1371/journal.pbio.2005188

**Published:** 2018-02-26

**Authors:** Yohann Boutté

**Affiliations:** CNRS-University of Bordeaux, UMR 5200 Membrane Biogenesis Laboratory, INRA Bordeaux Aquitaine, Villenave d’Ornon, France

## Abstract

Lipids are essential components of biological membranes that present a wide diversity in eukaryotic cells. Recent impressive advances in lipid biochemistry and biophysics have enabled a refocus of our view of lipids as functional units for cellular activity. However, the gap between molecular and cellular processes remains to be bridged. Here, 2 papers meet the burden of proof that choline transporters participate in local lipid composition modifications at the *trans*-Golgi network, an intracellular compartment that serves as the main sorting station in the cell. Localization of choline transporters to this precise compartment could be a way for plant cells to quickly modify the membrane lipid composition and asymmetry during both the allocation of cargos and the recruitment of trafficking machineries into distinct subcellular pathways.

## Choline is known as a precursor of the neurotransmitter acetylcholine but is also involved in phospholipid synthesis

Choline is an essential micronutrient that can also be considered to be a vitamin to the extent that the human body is not able to synthesise it in a sufficient quantity to ensure good health. Choline is a precursor for the neurotransmitter acetylcholine, which is used by neurons to communicate within the central and peripheral nervous systems. Choline transporters are localised at the cell surface of neurons and provide an entry checkpoint for choline in the cell. Hence, choline uptake is largely dependent on the quantity of choline transporters present at the plasma membrane (PM). This quantity is regulated by the internalisation of choline transporters from the PM to internal membrane compartments called endosomes, which can, when needed, fuse back to the PM. This process, known as “PM recycling,” allows adaptation of the quantity of intracellular choline to the quantity of neurotransmitters released. While neurons express mainly high-affinity choline transporters (CHTs), other choline transporters such as intermediate-affinity choline transporter-like (CTL) are ubiquitously expressed in human tissues as well as in other organisms, including plants [1–4]. CTLs were discovered only recently in 2000 [5], but in the last decade, experimental evidence brought forth more information on their expression patterns and their potential functions. In humans, there are at least 6 CTLs, all undergoing alternative splicing during their expression. In rat cells, it has been shown that pharmacological inhibition of CTL-mediated choline uptake results in abnormal lung surfactant [6]. Other studies support a role for CTL-mediated choline transport in lung and colon cancers [7]. Interestingly, the CTL4 protein of human cells is involved in acetylcholine synthesis in non-neuronal lung and colon cancer cells, while CTL1, 2, 3 and 5 do not play any role in this process [7]. Consistently, in the mouse trachea, CTL-mediated acetylcholine release has also been proposed as a process to activate trachealis muscles [8]. What could be the role for the CTLs that are not involved in acetylcholine transport? Several studies revealed that these medium-affinity choline transporters supply choline for phospholipid biosynthesis through the well-known cytidine-diphospho(CDP)-choline pathway that was discovered in the 1950s’ by Eugene Kennedy and thus also referred to as the Kennedy pathway [9]. However, although a link between CTLs and phospholipid biosynthesis has been established, the number of studies addressing CTL function has remained astonishingly low as compared to what is known for CHTs. Intracellular localisation of CTLs and choline-mediated lipid homeostasis, as well as the impact of CTLs on membrane lipid composition, trafficking pathways, and cellular processes, remain unclear. Moreover, CTLs have also been identified in plants, which do not possess a nervous system but are still able to produce acetylcholine and in which phosphatidylcholine (PC) is also an important membrane phospholipid. However, the number of studies on choline transporters in plants has remained extremely limited.

## Choline transporters: Little is known despite their physiological importance

In the plant model *Arabidopsis thaliana*, 7 CTLs have been identified; one of them is called CHER1/AtCTL1. In the *Arabidopsis* root, CHER1 is localised at the division plane in dividing cells, which can be due to the fact that CHER1 localises in intracellular punctuate structures that were identified as being part of the *trans*-Golgi network (TGN), a post-Golgi compartment serving a central station where the sorting of both PM recycling proteins and de novo synthesised proteins occurs [10]. In *Arabidopsis*, TGN-derived vesicles fuse at the division plane, in the middle of the cell to promote centrifugal extension of the division plane. Hence, CHER1 localisation at the division plane could be circumstantial given that when the division plane is fusing with the PM of the mother cell, CHER1 proteins disappear from the division plane, which becomes the new PM. However, one striking observation was that CHER1/CTL1 localised to specialised plant cells involved in long-distance transport of solute molecules. These cells, called “sieve tube elements,” are cylindrical and elongated cells transporting the elaborated sieve from the organs generating photosynthetic products to the organs consuming them. Sieve tube elements pile up to form phloem vessels that create a frame for the entire plant. More precisely, CHER1/CTL1 localises in a polar fashion on the side of the sieve tube element in contact with another sieve tube element [4]. The extracellular matrix (called the cell wall in plants) located between 2 sieve tube elements is known as the sieve plate and is actually perforated by sieve pores connecting the 2 cells to each other. In other tissues, these nanopore structures are named plasmodesmata (PD) and ensure PM continuity between adjacent cells and transport of products that transit from one cell to another through these membrane structures that enable symplastic connectivity. Analyses of a *ctl1*^*cher1*^ mutant of *A*. *thaliana* (point mutation causing a premature stop codon) have revealed the involvement of the choline transporter in phloem patterning and conductivity as well as PD formation and development [4,11]. A comparative proteomic profiling of PD-enriched biochemical fractions from the *ctl1*^*cher1*^ mutant identified a depletion of proteins containing C2 lipid-binding domains [12]. C2 lipid-binding domains proteins share topology similarities with synaptotagmins known to be involved in tethering of endoplasmic reticulum (ER)-PM contact sites [13]. Hence, CTL1 could be involved in phospholipid homeostasis at PD by regulating the synthesis of PC, which could in turn be a recruiter for C2 lipid-binding domains proteins, putatively important for PD ultrastructure.

## What is new about choline transporters?

The study from Gao and collaborators started with the identification of a new mutant, called *sic1* (for *Significant Ionome Changes1*), altered in ion homeostasis [14]. A map-based cloning revealed that this mutant was a new point mutation allele of *CTL1*. Ions are well known to be transported through PDs of the root cortex cells [15]. Hence, Gao et al. characterized the PD ultrastructure in the root cortex cells of the *ctl1*^*sic1*^ mutant and observed that PDs were shrunken in comparison with wild-type cells [14]. Moreover, the plasmodesmata callose-binding proteins 1 and 2 (PDCB1 and PDCB2, which are involved in regulation of callose deposition at PDs and the diffusion of molecules through PDs) are mislocalised from PDs to intracellular compartments in the *ctl1*^*sic1*^ mutant [14]. Conjointly, PD-mediated movement of the transcription factor SHORTROOT (SHR) from the stele to the endodermis layer was altered in the *ctl1*^*sic1*^ mutant, revealing a functional alteration of PD [14].

The other study from Wang and collaborators, published at the same time, identified leaf and hypocotyl cell elongation phenotypes in another mutant allele of *CTL1* [16]. This mutant allele is a transfer DNA (T-DNA) Salk insertion line and was previously known as the *cher1-4* mutant allele [4]. The study from Wang and collaborators identified additional phenotypes in the *ctl1*^*cher1-4*^ mutant such as apical hook development defects and reduced hypocotyl phototropic response to unilateral light [16]. The authors could show a misdistribution of the phytohormone auxin in the *ctl1*^*cher1-4*^ mutant during these physiological processes. Moreover, the *ctl1*^*cher1-4*^ mutant was found to be more resistant to external application of high auxin concentration [16]. Previously, in 2014, an elegant study used the hypocotyl phototropic response as a model system to show that PDs are involved in symplastic auxin movement and auxin redistribution during phototropism [17]. PD-mediated auxin diffusion was an important discovery and added another layer of complexity to the understanding of auxin homeostasis regulation during plant development and adaptation. However, are all phenotypic defects observed in *ctl1* mutant alleles attributable to PD defects?

Experimental evidences from both studies argue against the sole involvement of PD but rather favour a synergic effect of PD and intracellular trafficking. Gao and collaborators visualize that the ion transporter NRAMP1, as well as PDCB proteins, was mislocalised to internal compartments corresponding to early endosomes (EEs) in the *ctl1*^*sic1*^ mutant instead of being localised at the PM [14]. Additionally, the recycling of NRAMP1 and the auxin efflux carrier PIN1, from the PM to EEs and back to PM, is altered in the *ctl1*^*sic1*^ mutant [14]. Finally, a choline treatment also alters the PM recycling of NRAMP1 and PIN1 [14]. Wang and collaborators also detected a decrease of PIN1 and another auxin efflux carrier, PIN3, at the PM in the *ctl1*^*cher1-4*^ mutant [16]. This reduction at the PM is correlated with a defect in PM recycling of PIN1 and PIN3 in the root and apical hook of the *ctl1*^*cher1-4*^ mutant [16]. Indeed, *CTL1* is expressed in all tissues where auxin is known to act (meristems, vasculature, lateral root primordia, and apical hook). Hence, auxin-related phenotypes in *ctl1*^*cher1-4*^ mutant are likely to be a combination of defects in PD-mediated diffusion of auxin and intracellular trafficking defects of auxin carriers. However, PD-mediated diffusion of auxin in the *ctl1*^*cher1-4*^ mutant was not addressed in the 2 studies. In the future, this could help in deciphering the respective contribution of PD versus endomembrane trafficking in CTL1-mediated auxin distribution. In actual fact, it is very likely that TGN will appear as a central unit anyhow given that the TGN-localized CTL1 controls intracellular trafficking of PDCB proteins, which are involved in the regulation of callose deposition at PDs and the diffusion of molecules through PDs [14]. Hence, it is expected that CTL1 controls trafficking rather than PD functionality directly.

## All roads lead to the TGN

A main platform for intracellular trafficking is the TGN, which in plants is discernible at the *trans*-most side of the Golgi apparatus and is constituted from a cloud of vesicles interconnected by membrane tubules [18]. Several publications indicate that the TGN is made of distinct subpopulations [18–23]. The secretory vesicle (SV) domain is labelled by the syntaxin SYP61 and the transmembrane protein ECHIDNA and plays a role in secretory trafficking sorting to either the vacuole or the PM [21,24]. The clathrin-coated vesicles (CCVs) are labelled by clathrin and also by the RAB-GTPase RAB-A2a and play a role in the recycling sorting from the TGN back to the PM [25]. Interestingly, Wang and collaborators show that CTL1 is localised to both subdomains of the TGN. What could be the role of CTL1 in TGN subdomains?

TGN subdomains have different lipid composition; SVs are enriched in sphingolipids and sterols as compared to CCVs or to the Golgi apparatus [23]. Both studies published in this issue characterised lipid composition defects in *ctl1* mutant alleles [14,16]. Both studies found that glycerolipids, including PC and phosphatidylethanolamine (PE), were reduced in absolute quantity in *ctl1*^*cher1-4*^ and *ctl1*^*sic1*^ mutants [14,16]. However, an additional effect on sphingolipid composition was observed; the *ctl1*^*cher1-4*^ mutant displayed an increase in some ceramide species [16]. Previous studies have shown that modification of the composition of the ceramide pool composition has an impact on PIN1 auxin carrier PM recycling via CCVs/RAB-A2a compartments, while modification of the acyl-chain length composition of the glycosyl inositol phosphoryl ceramide (GIPC) pool of sphingolipids has an impact on PIN2 polar secretory sorting to apical PM via SVs/SYP61 compartments [23,26]. Hence, different sphingolipid pools could have different effects at different TGN subdomains. Interestingly, the *ctl1*^*cher1-4*^ mutant targets PIN1 and PIN3, but not other PM proteins, including the auxin carriers PIN2, AUX1, and LAX3 and the brassinosteroid receptor BRI1 [16]. These results indicate a specificity of trafficking substrates and fit well with a role of ceramide species in PIN1 PM recycling via CCVs/RAB-A2a compartments. However, CTL1 is reported to locate at both SVs and CCVs, and the link between choline and sphingolipids is not clear [16]. Since sphingomyelin (a ceramide with a PC or PE head group) has never been detected so far in plants, choline should act on sphingolipids in an indirect way that remains to be characterised during protein sorting at TGN.

**Fig 1:**
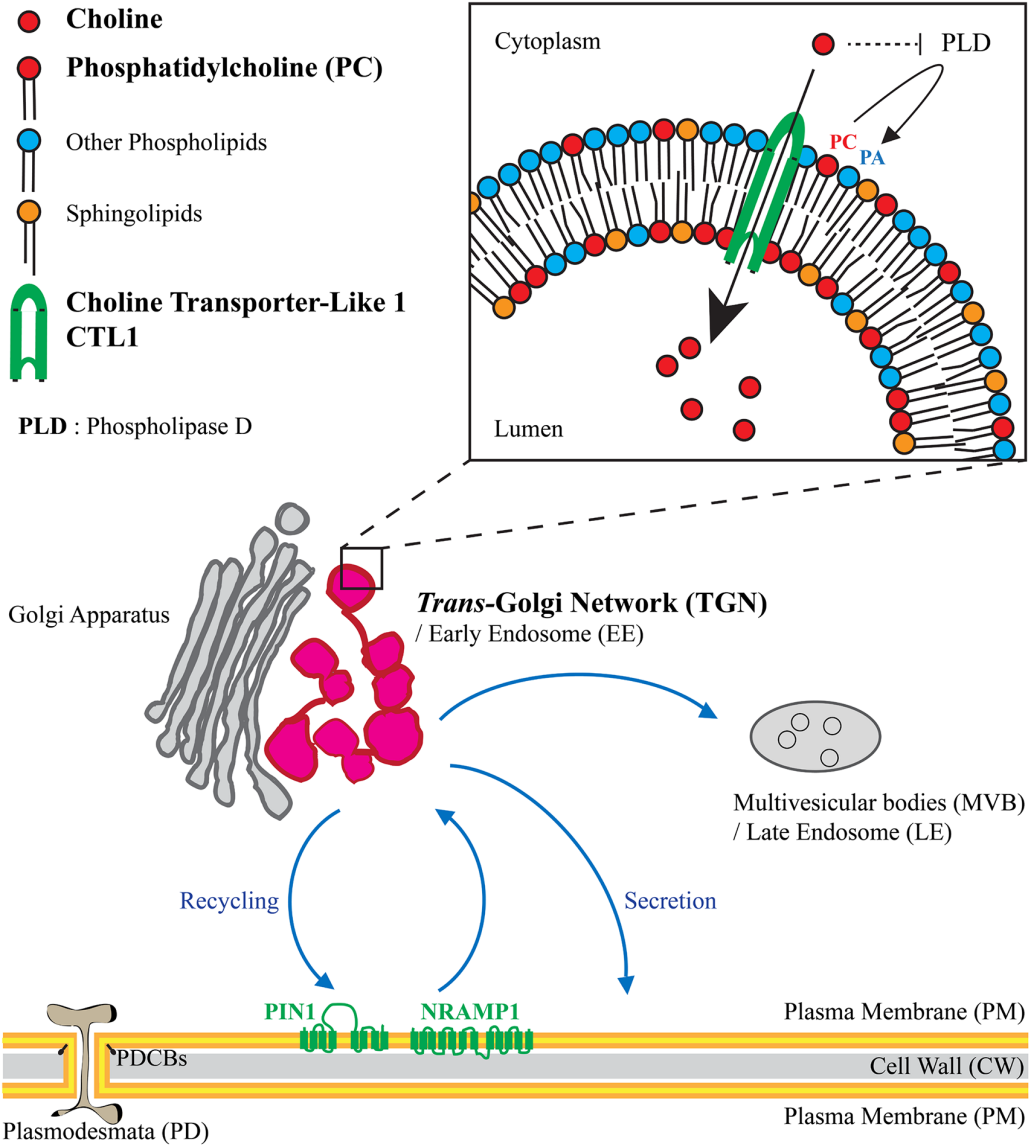
Choline transporter CTL1 locates at the *trans*-Golgi network (TGN), a post-Golgi compartment acting as a central hub in the cell where endosomal plasma membrane recycling takes place as well as secretory sorting to plasma membrane and trafficking to late endosomes. CTL1 is involved in phosphatidylcholine (PC) membrane homeostasis, probably by creating a gradient of choline on either side of TGN membranes. Two studies published in this issue of *PLOS Biology* show that TGN sorting of plasmodesmata callose-binding proteins (PDCBs), the ion transporter NRAMP1, and the auxin carrier PIN1, which polarly localised at the basal plasma membrane, depends on choline-mediated lipid homeostasis. Abbreviations: PA, phosphatidic acid.

This being said, Gao and collaborators explored a different hypothesis. They reasoned that because both PC and PE are substrates for phospholipase D (PLD)—to produce phosphatidic acid (PA)—and because the *ctl1*^*sic1*^ mutant is more resistant to PLD inhibitors, free choline could inhibit PLD activity. Together with their finding that a high concentration of choline inhibits vesicle trafficking, they argue for a hypothesis in which choline is sequestrated in TGN vesicles to maintain a low PC/PA ratio at the cytoplasmic leaflet of TGN vesicles because of cytosolic PLD activity. By contrast, a higher PC/PA ratio could be present at the luminal leaflet of membrane vesicles (Fig 1). This hypothesis is very attractive because of the driving force that the physical properties of lipids confer to membrane morphodynamics [27]. However, this lipid asymmetry between the 2 leaflets of TGN vesicles would require further experimental investigations in the future.

## Conclusion and perspectives

There is no doubt that asymmetry in lipid composition between 2 leaflets of a given membrane exist; this was first discovered in the PM of human erythrocytes, but this phenomenon is likely a universal process that also occurs at TGN-associated vesicles [28]. Understanding the amazingly wide nature and function of lipid diversity in membrane trafficking is an immense challenge. Membrane trafficking is extremely dynamic, and remodelling of the lipid composition of vesicles is constantly occurring and serves as a platform for protein sorting and trafficking machineries that will define the nature of a vesicle and its fate inside the cell. In plants, the TGN is highly complex, made of subdomains fulfilling different functions, e.g., sorting of secretory cargos and cargos coming from the recycling/endosomal pathway; it has also been shown that the late endosomes (LEs, which are equivalent to the multivesicular bodies [MVBs]) already mature from the TGN [29]. Hence, the TGN is a place where lipid remodelling is expected to be highly dynamic and compartmentalised so that secretion, recycling, and late endosomal pathways can be differentiated. Technical advances have definitely breathed new life into our understanding of TGN subdomain composition both by proteomic and lipidomic profiling using immunoprecipitation that has proved to yield high purification of TGN subdomains with a high level of separation. Chemical genomics is undeniably combinatory to classical genetic approaches that have clear limitations in answering the dynamics of the TGN subpopulation. Super-resolution live-imaging techniques will help us to get a clearer view on that issue, but still, the dream would be to visualise and track lipid homeostasis, asymmetry, and dynamics directly in living cells that are as close as possible to their native state. The realisation of this dream would in effect crack the major present limitation of the field.

## Abbreviations

CCV: clathrin-coated vesicle
CDP: cytidine-diphosphate
CHT: high-affinity choline transporter
CTL: choline transporter-like
EE: early endosome
ER: endoplasmic reticulum
GIPC: glycosyl inositol phosphoryl ceramide
LE: late endosome
MVB: multivesicular body
PA: phosphatidic acid
PC: phosphatidylcholine
PD: plasmodesmata
PDCB: plasmodesmata callose-binding protein
PE: phosphatidylethanolamine
PLD: phospholipase D
PM: plasma membrane
SHR: SHORTROOT
SV: secretory vesicle
T-DNA: transfer DNA
TGN: *trans*-Golgi network
